# Lung Infections in Systemic Rheumatic Disease: Focus on Opportunistic Infections

**DOI:** 10.3390/ijms18020293

**Published:** 2017-01-29

**Authors:** Manuela Di Franco, Bruno Lucchino, Martina Spaziante, Cristina Iannuccelli, Guido Valesini, Giancarlo Iaiani

**Affiliations:** 1 Rheumatology Unit, Department of Internal Medicine and Medical Specialties, Sapienza University of Rome, Rome 00161, Italy; lucchino.b@gmail.com (B.L.); cristina.iannuccelli@uniroma1.it (C.I.); guido.valesini@uniroma1.it (G.V.); 2 Tropical Medicine Unit, Department of Infectious Diseases, Azienda Ospedaliera Universitaria Policlinico Umberto I, Rome 00161, Italy; martinaspaziante@hotmail.com (M.S.); giancarloiaiani@gmail.com (G.I.)

**Keywords:** rheumatic diseases, lung infections, opportunistic infections, anti-tumor necrosis factor (TNF), pneumonia

## Abstract

Systemic rheumatic diseases have significant morbidity and mortality, due in large part to concurrent infections. The lung has been reported among the most frequent sites of infection in patients with rheumatic disease, who are susceptible to developing pneumonia sustained both by common pathogens and by opportunistic microorganisms. Patients with rheumatic disease show a peculiar vulnerability to infectious complications. This is due in part to intrinsic disease-related immune dysregulation and in part to the immunosuppressive treatments. Several therapeutic agents have been associated to a wide spectrum of infections, complicating the management of rheumatic diseases. This review discusses the most frequent pulmonary infections encountered in rheumatic diseases, focusing on opportunistic agents, consequent diagnostic challenges and appropriate therapeutic strategies.

## 1. Introduction

Systemic rheumatic diseases are characterized by considerable morbidity and mortality due to disease complications and, particularly, concurrent infections [1]. Respiratory tract infections are common in patients with rheumatic disorders. In these patients, pneumonia can be sustained by common pathogens, like bacteria and viruses, and by opportunistic infections. The latter are emerging as an important cause of death in developed countries [2]. The main causes of vulnerability in patients with rheumatic disease are represented by alterations of immunoregulation, disease severity, debility, comorbid illnesses, and the use of immunosuppressive medications [3]. Immunosuppressive therapy and biological agents, especially when used in combination, have been reported to increase risk of infection by opportunistic pathogens such as *Mycobacterium* spp., *Pneumocystis jiroveci*, *Histoplasma capsulatum*, *Coccidioides immitis*, *Aspergillus* spp., and *Nocardia* spp., as well as routine bacterial and viral respiratory pathogens [4,5,6].

## 2. Susceptibility to Infections in Systemic Rheumatic Diseases

Patients affected by systemic rheumatic diseases show an intrinsic predisposition to infections. These can be associated to three main factors:

- Immunological factors
- Disease related factors
- Drugs related factors

### 2.1. Immunological Factors

There are several immunological alterations in rheumatic diseases that may cause predisposition to infective risk. One of the major alterations is represented by impairment of the complement system. In fact, deficiency of complement factors is strongly associated to the development of systemic lupus erythematosus (SLE) and to an increased susceptibility to infection by encapsulated bacteria (*Streptococcus pneumoniae* and *Neisseria Meningitidis*) [7]. Furthermore, deficiency of the complement receptors, described in erythrocytes, lymphocytes and neutrophils from SLE patients, may also interfere with complement-mediated microorganism clearance [8]. Evidence of immune dysregulation has been reported also in rheumatoid arthritis (RA). In chronic inflammatory conditions such as RA, persistent antigen stimulation results in T cell homeostasis alteration, with oligoclonal expansion of peripheral T cells and a global reduction of the T cell receptor (TCR) repertoire. These oligoclonal T cells can persist for years, competing for growth factors and space, resulting in a severe contraction of the T cell pool functionally available to react against foreign antigens [9].

### 2.2. Disease Related Factors

Susceptibility to infection can be associated to the organ damage due to systemic rheumatic diseases. Organ damage can lead to the development of a “locus minoris resistentiae”, vulnerable to superimposed infections. A cohort study on idiopathic pulmonary fibrosis patients with acute exacerbation of respiratory symptoms requiring hospitalizations showed the presence of an infectious agent virtually able to cause the sudden decline of the respiratory function in about one-third of cases [10]. Infections in pulmonary fibrosis secondary to systemic rheumatic diseases are well described. In a multivariate analysis of an RA patient cohort, the presence of an underling interstitial lung disease (ILD) and rheumatoid nodules were strong predictors of infection [11]. Esophageal involvement, like dysmotility disorder or esophageal reflux, can also be associated to pulmonary infections. In systemic sclerosis (SSc), the most frequent causes of death not directly related to SSc are infections, especially “ab ingestis” pneumonia [12].

### 2.3. Drugs Related Factors

The frequent use of immunomodulatory/immunosuppressive drugs exposes rheumatic patients to a significantly raised risk of infections. The drugs used in rheumatic disease can be divided into glucocorticoids, conventional disease-modifying anti-rheumatic drugs (DMARDs) and biological DMARDs. Glucocorticoids (GC) are widely used in treatment of rheumatic diseases, showing anti-inflammatory and immunosuppressive properties. GC treatment can plausibly increase the risk of infections interfering with phagocyte function and suppressing cell-mediated immunity [13]. A meta-analysis of over 2000 patients randomly allocated to systemic GC treatment in the setting of different diseases showed a higher risk of overall infections in treated patients, with no differences between low or high dose [14]. In a large cohort of RA patients, prednisone use was found to be a significant independent predictor of hospitalization for pneumonia, with a dose-related increase of risk [15]. Similarly, in SLE patients treated with GC, a higher rate of fungal infections and a higher mortality have been reported [16]. GC treatment in SLE patients and in patients affected by granulomatosis with polyangiitis has also been associated with a raised risk of developing *Pneumocystis* pneumonia [17,18]. Considering the influence of GC on infection risk, the European League Against Rheumatism (EULAR) recommends evaluating and eventually treating chronic or recurrent infections prior to starting treatment with GC and during therapy [19]. Therapy with conventional DMARDs (cDMARDs) represents an additional factor of increased risk of infections. The risk varies depending on the different cDMARDs used. In a large cohort of RA patients, cyclophosphamide was associated with the highest risk of severe infections requiring hospitalization, while azathioprine was associated to a moderate increase of risk. Methotrexate was found to moderately increase the risk of hospitalization for pneumonia. Conversely, antimalarial agents, leflunomide, sulfasalazine, cyclosporine, and other DMARDs were not associated with a raised risk of infections [20]. Nevertheless, according to the complexity of rheumatic patients and the frequent combinations of different classes of DMARDs, a tight control to promptly identify infections is advisable, independent of DMARD class [21]. Anti-tumor necrosis factor (TNF) agents have emerged as the treatment of choice in many rheumatic diseases, primarily aggressive forms of RA. TNF inhibition can lead to infection or reactivation of granulomatous infections like tuberculosis and fungal infections, such as *Histoplama*, *Coccidioides*, *Aspergillus*, *Pnemocystis* and *Nocardia.* The impaired macrophage-killing capacity can also facilitate bacterial infections, like *Pneumococcus* or *Legionella* pulmonary infections as well as disseminated infections by *Salmonella* or *Listeria.* Although less frequent, patients treated with anti-TNF agents can develop invasive viral infections sustained by varicella-zoster virus or cytomegalovirus, as occur in immunosuppressed patients [4]. Rituximab (RTX), an anti-CD20 antibody that causes a profound depletion in B cell populations, is widely used in the treatment of systemic rheumatic diseases. Currently, RTX is approved in RA and anti-neutrophil cytoplasmic antibody (ANCA)-associated vasculitis, and is frequently used for off-label treatment of many refractory diseases, like SLE with renal or central nervous system involvement, SSc, and Sjogren’s syndrome [22]. Many reports showed a raised incidence of infections in patients treated with RTX. Several potential mechanisms have been proposed to explain the increased rate of infections during RTX therapy. Neutropenia and hypogammaglobulinemia, occurring frequently during repeated administrations of RTX, have been linked to an increased incidence of overall infections [23,24]. The depletion in B cells performed by RTX, decreasing the ability to develop a humoral response to new antigens and the antigen presenting cell function of B cells, has been associated to an impaired ability to react to mycobacterial and *Pneumocystis* infections [25,26]. However, to date there is inconclusive evidence of a raised risk *Pneumocystis* or mycobacterial infections in RTX-treated patients. In spite of this, caution in patients treated with RTX is advisable, through watchful clinical and laboratory monitoring [24]. Table 1 summarizes most frequent infections associated with immunosuppressive drugs.

## 3. Lung Infections in Systemic Rheumatic Diseases

### 3.1. Lung Infections in Rheumatoid Arthritis

Lung infections are a major cause of death in RA. In RA patients, lung infections might be facilitated by impaired immunity, immunosuppressive treatment, pre-existent interstitial lung disease (ILD) or other kinds of lung involvement by RA [27,28].

#### 3.1.1. Pneumonia in Rheumatoid Arthritis

Infectious pneumonia sustained by usual pathogens is among the most frequent infections in RA patients. In a large RA patient cohort of over 16,000 patients, the incidence of common pneumonia was 17 cases per 1000 patients-year. Factors associated with raised risk of pneumonia were diabetes, smoking, prior myocardial infarction and pre-existent pulmonary disease. Pneumonia risk was also influenced by RA disease duration and by performance status measured by the Health Assessment Questionnaire score. The latter was also the most powerful predictor for hospitalization. Prednisone was strongly associated to pneumonia risk in a dose-dependent manner, while leflunomide was associated with a slightly increased risk. No increased risk was found with anti-TNF therapy or methotrexate [15].

#### 3.1.2. Infectious Complications of Rheumatoid Arthritis with Lung Involvement

Pulmonary involvement in RA can present various manifestations, including parenchymal, pleural, airways and vascular involvement. Some of these manifestations, like rheumatoid nodules and bronchiectasis, can involve complications with superimposed infections. Rheumatoid nodules, a characteristic lesion frequently founded in patients with longstanding RA, can occur in the lung [29]. Accelerated lung nodulosis is a well-known complication of RA treatment with methotrexate, leflunomide and, possibly, anti-TNF agents [30,31,32]. Lung rheumatoid nodules are most often asymptomatic unless they cavitate or rupture. In these cases, superinfection, pleural effusion or bronchopleural fistula may occur [33]. Bronchiectasis is frequently founded in RA patients, with a prevalence of 18%–30% on chest high-resolution computed tomography [34]. RA patients with bronchiectasis show a mortality 7.3 times higher than the general population and 5 times higher than RA patients without bronchiectasis. The majority of deaths are due to lung infections [35]. In RA patients with bronchiectasis, biologic DMARD treatment and sputum colonization by pathogenic bacteria, like *Pseudomonas aeruginosa*, *Staphylococcus aureus* or *Haemophilus Influenzae*, have been identified as predictive risk factors for pneumonic complications. In patients with definite bronchiectasis, a careful assessment of infective risk should be performed before biologic prescription [36].

#### 3.1.3. Anti-TNF Treatment and Lung Infections in Rheumatoid Arthritis

**Figure 1:**
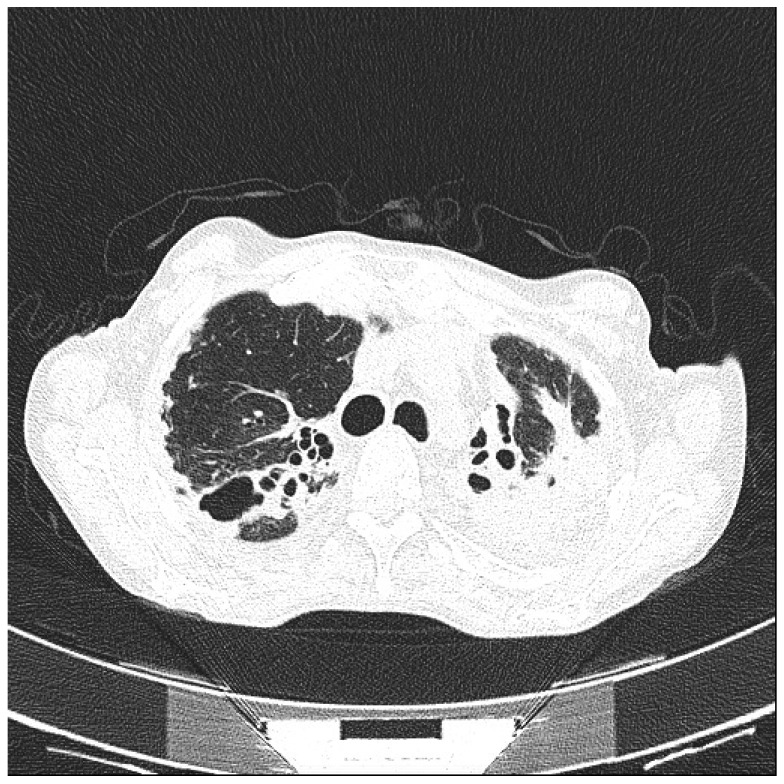
Advanced pulmonary tuberculosis in a patient with rheumatoid arthritis.

Biologic DMARDs, especially anti-TNF agents, are a further risk factor for many opportunistic infections. Interfering with the involvement of TNF on granuloma formation, anti-TNF agents increase the risk of developing tuberculosis (TB) (Figure 1). Compared to the general population, TB risk is increased by 2- to 10-fold in TNF antagonist-naïve RA patients and by 2- to 30-fold in TNF antagonist-exposed RA patients [37,38,39]. However, in the last decades, a significant reduction of TB has been observed, supposedly related to the introduction of systematic screening procedures before starting biologic therapy. Most of the active TB cases in patients treated with TNF antagonists are due to reactivation of latent TB infection. Once developed, the disease shows rapid progression and dissemination. The risk associated to anti-TNF agents is limited to the first 5 years of therapy. This particular timing could be related to the effect of anti-TNF on latent bacilli [40,41]. Nevertheless, patients treated with anti-TNF agents are exposed to newly acquired TB infections. Furthermore, anti-TNF therapy exposes RA patient to a raised risk of other granulomatous infections caused by various bacteria or fungi like *Candida*, *Aspergillus*, *Coccidioides*, *Histoplasma*, *Nocardia*, *Listeria* and nontuberculous mycobacteria. Therefore, close monitoring during the treatment course is advisable [42,43].

### 3.2. Lung Infections in Systemic Lupus Erythematosus

Despite a general increase of survival rate of SLE patients in recent years, infections remain a major cause of death in SLE worldwide [44]. The lung has been reported as the most frequent site of infections in SLE patients, and prednisone and immunosuppressive drugs use have been identified as strong predicting factors for infection development [45].

#### 3.2.1. Bacterial Pneumonia in Systemic Lupus Erythematosus

Bacteria represent most frequent etiological agents of pneumonia in SLE patients. The most common pathogens are *Staphylococcus aureus*, *Streptococcus pyogenes*, *S. pneumoniae* and *Escherichia coli*, although hospital-acquired infections can be sustained by multi-drugs resistant bacteria, such as *Pseudomonas.* Several factors, like chemotaxis and opsonization defects, splenic dysfunction and hypocomplementaemia cause SLE patient susceptibility to develop infections sustained by encapsulated bacteria. *S. pneumoniae* is the leading cause of pneumonia in SLE patients. In SLE patients, pneumococcal pneumonia is often severe, frequently acquiring a septicemic and fulminant course, occasionally described even at disease presentation [46,47,48,49,50]. For these reasons, a polyvalent pneumococcal vaccine is strongly recommended in SLE patients [51].

#### 3.2.2. Mycobacterial Infections in Systemic Lupus Erythematosus

The rate of tuberculosis (TB) is higher in SLE patients, with a 7-fold higher incidence of TB compared with the general population in developed countries and an estimated prevalence of 5% in developing countries, often with extrapulmonary features [52,53]. SLE patients are also susceptible to non-tuberculous mycobacteria (NTM) infections, although less frequently. Lung infections follow soft-tissues and skin localization in NTM infections, and show a lower tendency to dissemination. NTM infections occur mostly in more immunosuppressed patients and treatment with high doses of GC seems to be the major risk factor [54].

#### 3.2.3. Cytomegalovirus Infection in Systemic Lupus Erythematosus

Among viral infections, cytomegalovirus (CMV) can represent the etiologic agent of pneumonia, sometimes in the context of severe multi-organ involvement. CMV infections in SLE patients can follow three possible patterns: CMV infections can induce the exacerbation of underlying SLE, triggering flares, or can result in a systemic viral infection with multi-organ manifestations, especially in most immunosuppressed patients. Finally, CMV infection can occasionally represent a trigger for the onset of SLE [55]. Lymphopenia, age older than 60 years and pulsed GC administrations have been reported as stronger risk factors for CMV opportunistic infections. In patients treated with aggressive immunosuppression and respiratory symptoms, possible opportunistic CMV pneumonia should always be considered [56].

#### 3.2.4. Fungal Infections in Systemic Lupus Erythematosus

In SLE patients, invasive fungal infections have been estimated to be the cause of hospital admission in about 0.6%–3.2% of cases. Mycotic infections are often severe, reaching an overall mortality range of 25%–70%. A significant association with high SLE activity and immunosuppressor use, especially high dose GC, azathioprine, cyclophosphamide and mycophenolate has been reported. Pulmonary aspergillosis is second in terms of frequency, following criptococcal meningitis [57]. In SLE patients, especially in cases of immunosuppression and neutropenia, pulmonary aspergillosis shows an invasive course, with tissue dissemination of mycotic hyphae. The diagnosis is difficult because 30% to 50% patients show no evidence of the fungus in blood and sputum cultures. Test for galactomannan, a polysaccharide of the *Aspergillus* cell wall, which can be performed on various biologic specimens, is a useful diagnostic tool [58,59]. *Pneumocystis jiroveci* pneumonia (PJP) is a fungal opportunistic infection unfrequently observed in profound immunosuppressed SLE patients. PJP usually presents as severe, bilateral interstitial pneumonia with insidious onset and high risk for requiring ventilator support. Mortality associated to PJP is high. Despite the lack of precise guidelines for prophylaxis of PJP in SLE patients, trimethoprim-sulfamethoxazole prophylactic treatment should be considered in SLE patients at higher risk of PJP [60,61].

### 3.3. Lung Infections in Systemic Sclerosis

In systemic sclerosis (SSc), the lung is a frequent site of disease involvement, both as primary manifestation of the disease and as target of secondary complications. Recent data from the EULAR Scleroderma Trials and Research (EUSTAR) database revealed that pneumonia was the most common severe infection in SSc patients. In the majority, pneumonia was associated to SSc-related esophageal reflux and documented aspiration [12]. Aspiration pneumonia develops after the inhalation of colonized oropharyngeal material in patients who present a disruption in gastro-esophageal junction integrity. The latter can result in oropharyngeal colonization by potential respiratory tract pathogens, including Enterobacteriaceae, *Pseudomonas aeruginosa,* and *Staphylococcus aureus.* The further presence of dysphagia increases the risk of aspiration and pneumonia development. Anaerobic bacteria may be found in the later course of the illness, frequently complicated by necrotizing pneumonia, abscess formations and empyema [62]. Up to 75% of SSc patients, even without symptoms suggestive of esophageal involvement, present signs of esophageal dysmotility at manometry or video-radiology studies [63,64]. Aspiration has been reported as the strongest risk factor for in-hospital mortality in SSc patients, even without pre-existing interstitial lung disease [65]. Furthermore, many drugs used in SSc treatment, such as cyclophosphamide, mycophenolate and rituximab have been associated to a raised risk of developing infections, mostly bacterial or mycotic pneumonia [66,67,68]. Thus, when patients with SSc manifest new pulmonary symptoms, especially if treated with potential armful immunosuppressive drugs, both routine and opportunistic lung infections should be considered for appropriate diagnostic and therapeutic intervention [69].

### 3.4. Lung Infections in ANCA-Associated Vasculitis

Anti-neutrophil cytoplasmic antibody (ANCA)-associated vasculitis (AAV; e.g., granulomatosis with polyangiitis, microscopic polyangiitis and eosinophilic granulomatosis with polyangiitis) presents as systemic small vessels vasculitis, which has historically been associated with high rates of disease-related acute mortality. Currently, the use of effective immunosuppressive strategies allows to obtain a remission in 80%–85% of patients affected by AAV, with an overall improvement of survival. However, immunosuppressive treatment has been associated with a high burden of infection-related mortality during the first 12 months. Data from the European Vasculitis (EUVAS) group, the largest AAV study reported to date, showed that 1-year overall mortality probability was 11%, and infections were the most common cause of death, accounting for half of all death observed [70]. In a large cohort of AAV patients, the 1-year incidence of at least one infection was 56%, with pulmonary and upper respiratory tract infections as the most common reported. The greatest number of infections occurred in first 3 months after treatment. Among causative organisms, *S. Aureus* was preponderant, followed by Gram-negative organisms and yeast. Only one case of *Pneumocystis* pneumonia was recorded. Therefore, considering the high incidence of infections after immunosuppressive therapy in AAV patients and the high burden of mortality associated to infections, prophylactic strategies specifically directed to the most common infections observable in AAV patients, including pneumonia, could decrease mortality [71].

### 3.5. Lung Infections in Sjogren’s Syndrome

Sjogren’s Syndrome (SS) is a chronic inflammatory disease characterized by lymphocyte infiltration of exocrine glands and destruction of the functional parenchyma, resulting in sicca syndrome. Moreover, many organs may be involved during SS, lungs included, with many patterns of possible interstitial lung disease such as nonspecific interstitial pneumonia, usual interstitial pneumonia, lymphocytic interstitial pneumonia and organizing pneumonia. Recurrent infectious pneumonia has been reported in about 10%–35% of SS patients [72]. Many possible mechanisms can explain the raised incidence of pneumonia in SS patients, for example reduced salivary production and the subsequent loss of its antibacterial effect, the frequent detection of periodontal disease and bronchiectasis, abnormalities in mucociliary clearance, and the higher incidence of esophageal reflux [73,74,75]. Finally, SS patients may be susceptible to developing common and opportunistic infections during immunosuppressive treatment for SS itself or for its complications such as lymphoma [76,77].

## 4. The Most Frequent Opportunistic Lung Infections and Their Management

### 4.1. Bacterial Infections

#### 4.1.1. Legionellosis

*Legionella pneumophila* is an intracellular Gram-negative bacteria that lives in aquatic environments and can cause mild to severe diseases, including community or hospital-acquired pneumonia (legionellosis). Legionella infection occurs through inhalation of contaminated aerosols produced by water systems such as hot water distribution systems, cooling waters or showers, being sometimes responsible for community-acquired outbreaks. Traditional risk factors for legionellosis include advancing age, male sex, cigarette smoking, recent surgery, chronic lung disease and immunosuppression, particularly GC therapy.

Recently, many cases of severe pneumonia have been reported in patients treated with anti TNF agents or other biological drugs as anti-interleukin-6 [78,79]. According to the French registry, the relative risk of Legionellosis has been estimated at 16.5–21 for patients with chronic inflammatory diseases receiving anti-TNF therapy [80]. Murine models demonstrated that TNF plays a protective effect versus *Legionella pneumophila* infections [81,82]. In facts, these bacteria have antigenic components, e.g. lipopolysaccharide, that strongly induce TNF production and consequent activation of cell-mediated immune response. Rats treated with anti-TNF agents and infected with *L. pneumophila* showed persistent pneumonitis with a higher bacterial load, impaired recruitment of peripheral blood monocytes to the lung and higher number of infected macrophages when compared to controls. Immunosuppression is associated with an increased severity of the disease, higher complication rates and higher mortality. Among this patient population, an increased risk of cavitation even in presence of appropriate antimicrobial therapy has also been demonstrated, probably due to sequestration of bacteria in necrotic tissue. Cavitation has been associated with *L. pneumophila* serotypes 1, 3, 4, 5, 6 and 8 as well as other non-pneumophila *Legionella* species: *L. micdadei*, *L. bozemanii*, *L. dumoffii* and *L. longbeachae*. Non-pneumophila species resulted to be more common in immunodepressed patients, accounting for 71% of all *Legionella* infections in this population. Bodro and collegues reviewed the literature describing 105 cases of *Legionella pneumonia* in patients treated with biologic therapies [83]. Sixty-four patients (65.3%) were treated with infliximab, twenty-three (23.5%) with adalimumab, five (5%) with etanercept and three (3%) with rituximab. The majority of patients received one or more associated immunosuppressive drugs, mainly GC (43%). Overall risk of infection demonstrated to be higher during the first 6 months of treatment. Patients treated with biologics can initially present with mild symptoms, such as general fatigue even in absence of fever [84]; dyspnea typically occurs after few days. Infection can occasionally result in inappropriate secretion of antidiuretic hormone, which produces hyponatremia. The clinicians should be aware about the disease in order to perform an early diagnosis. In clinical practice, urine assay for *Legionella* antigen is routinely used, being rapid and specific for *L. pneumophila* serotype 1, with a sensitivity of 70%–90% and specificity approaching 100%. However, if a non-pneumophila species is clinically suspected, a molecular assay by PCR on culture of respiratory tract specimen should be performed. Clinical usefulness of serology is limited because of the reduced sensibility rates in immunosuppressed patients. Appropriate and prompt antimicrobial therapy is essential; agents active against Legionella species include macrolides, quinolones, tetracyclines and rifamycins. In presence of cavitation, 14-day long-term treatment should be used. In case of severe disease, intravenous moxifloxacin or levofloxacin and azithromycin have been proposed, eventually administered in addition to rifampicin.

#### 4.1.2. Tuberculosis

In order to understand the reasons underlying the increased risk of TB development among patients treated with anti-TNF drugs, it is necessary to analyze mechanisms of immune response to *Mycobacterium tuberculosis*. *M. tuberculosis* bacilli are transmitted via the respiratory route. In the lung, they interact with macrophages, initiating an inflammatory response via cytokine cascade (TNF, IL-12, IL-1, IL-6) that induces migration of other immune cells to the lung [39]. Under the influence of cytokines, recruited monocytes differentiate into macrophages that, together with dendritic cells, engulf mycobacteria. Subsequently, engulfed macrophages migrate to the regional lymph nodes [85] where they activate CD4 and CD8 T cells that travel back to the lung where they cause granuloma formation. CD4 and CD8 T cells are essential in the immune response to *M. tuberculosis*, through production of cytokines as IFN-γ and TNF. It is known that the release of TNF in response to mycobacterial infection has several beneficial effects. In vitro studies showed that TNF increases the ability of macrophages to phagocytose and kill mycobacteria and other intracellular pathogens via both nitric oxide-dependent and nitric oxide-independent pathways [86]. TNF plays an essential role in control and containment of intracellular pathogens, recruiting inflammatory cells to the area of infection and stimulating the formation and maintenance of granulomas that physically contain infection [87]. Thus, treatment with TNF antagonists can cause reactivation of latent tuberculosis infection (LTBI) leading to pulmonary, extrapulmonary or disseminated disease. Kaneko et al demonstrated that in TNF-knockout mice infected with *M tuberculosis*, the survival time decreased from 50 to 33 days [88]. Diffuse abscesses developed in the lungs, liver, spleen, and kidneys, without typical granulomas. Every six months, pharmaceutical companies provide periodic safety updates (PSUR) based on clinical trials, follow-up, and post-marketing reports [89]. Recent data show that TB is the most common opportunistic infection associated to anti-TNF treatment (0.06%) [90,91]. In PSUR-6, the disease was more common in patients with RA (62.1%) than with Crohn’s disease (20.2%). Pulmonary TB was the most common form of disease (69.1%) while miliary TB was less common (20.6%). The presence of fever, night sweats or weight loss, even if not specific, should increase suspicion of TB. Chronic cough and haemoptysis suggest pulmonary tuberculosis. Atypical presentation could result in diagnostic delay. Clinicians should be aware since disseminated disease constitute a significant, although minor, part of the total cases [89]. In patients treated with anti-TNF agents, TB occurs mostly after few administration of the drug, probably as consequence of drug-induced reactivation of latent bacilli in unknowingly previous exposed individuals. Recently, clinicians became more aware about TB risk in this population. In fact, even if the number of patients treated with anti-TNF continues to increase, the number of cases of TB assessed by PSUR-6 shows a decline, reflecting the institution of mandatory purified protein derivative testing and chest X-ray prior to the administration of therapy as well as earlier diagnosis. For the same reason, the overall TB fatality rate in PSUR-6 declined in the last years. Before starting anti-TNF therapy, an accurate history of prior TB infection should be collected and a careful physical examination, chest radiograph, and tuberculin skin test or IFN-γ release assay (IGRA) should be performed [91]. Patients with diagnosis of active TB (either pulmonary or extrapulmonary) prior to starting anti-TNF therapy should be treated with standard four-drug therapy (isoniazid, rifampicin, ethambutol and pyrazinamide). A minimum waiting period of 2 months of antimycobacterial treatment is necessary before starting anti-TNF therapy. Ideally, anti-TNF therapy should be delayed until anti-TB treatment is completed. Patients with a screening positive purified protein derivative or IGRA test but normal chest radiograph should receive prophylaxis with isoniazid treatment for 6 months, or isoniazid plus rifampin for 3 months before receiving anti-TNF treatment [89,92,93]. In patients with an abnormal screening chest radiograph, active TB should be ruled out. If active TB has been excluded, chemoprophylaxis with isoniazid for 6 months is suggested, four weeks before initiating anti-TNF therapy. Patients with history of prior pulmonary or extrapulmonary TB, who have received adequate treatment, can start anti-TNF therapy but they should be closely monitored with a chest radiograph every 3 months. Patients who develop active TB (either pulmonary or extrapulmonary) while on anti-TNF therapy should be treated with standard four-drug therapy, and the anti-TNF therapy can be continued if indicated [4].

### 4.2. Fungal Infections

#### 4.2.1. Pneumocystosis

The incidence of *Pneumocystis jiroveci* pneumonia (PJP) among patients on TNF inhibitors varies between < 0.01/1000 patients/year in North America to 8.8/1000 patients/year in Japan [94] due to the different testing modalities as well as differences in analyzed population. In 2009, Arnold and coll. demonstrated that up to a quarter of patients treated with anti-TNF were asymptomatically colonized by PJ. Among symptomatic patients, a shorter duration of symptoms (∼1 week), lower β-d glucan and higher C-reactive protein levels were documented [95,96]. Mu and coll. defined different radiological stages of PJP in non- acquired immunodeficiency syndrome (AIDS) immunocompromised patients. The manifestations of chest X-ray and chest computed tomography (CT) of PJP were both divided into three stages: early, mid, and late stage. In early stage, chest X-ray was normal or nearly normal while chest CT scan showed bilateral diffuse ground-glass opacities; in mid stage, chest X-ray documented bilateral pulmonary infiltrates while chest CT scan showed bilateral diffuse ground-glass opacities and patchy consolidations; in late stage, chest X-ray showed bilateral pulmonary consolidations while chest CT scan showed bilateral predominant consolidations. However, the stages of chest X-ray and chest CT are not certainly accordant with each other. The fatality rate of all patients was 34.3%. At first chest X-ray, eighteen cases were at early stage and fatality rate was 0%, fifty cases were at mid stage and fatality rate was 28%, and thirty-seven cases were at late stage and fatality rate was 59.5% [97]. There is no worldwide consensus on prophylaxis for PJP. In North America and Europe, routine prophylaxis is likely unnecessary while in Japan patients have shown some benefit with trimethoprim/sulfamethoxazole prophylaxis. In particular, this should be considered for patients treated with both high-dose corticosteroids and TNF inhibitors [96].

#### 4.2.2. Aspergillosis

**Figure 2:**
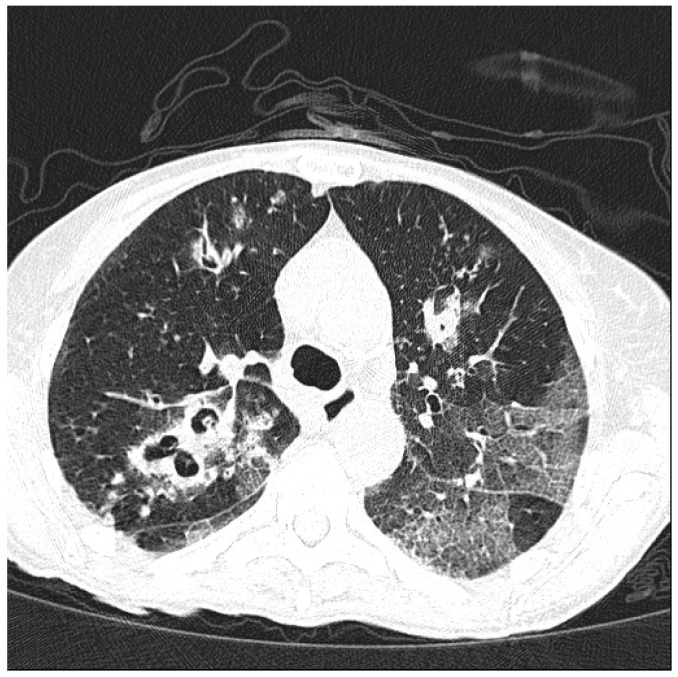
Pulmonary invasive *Aspergillosis* in a patient with anti-neutrophil cytoplasmic antibody (ANCA)-associated vasculitis.

*Aspergillus* spp. are ubiquitous in environment and the infection is usually transmitted via inhalation of infectious conidia. Invasive aspergillosis (IA) is an opportunistic infection most commonly seen in patients with prolonged neutropenia [95]. Rarely the infection can occur through direct skin inoculation or the gastrointestinal tract. *Aspergillosis* can cause respiratory tract diseases, cutaneous infection, or extrapulmonary dissemination. Disseminated disease is most frequently associated with immunocompromised states (e.g., patients with history of recent hematopoietic cells or solid organ transplantation, hematologic malignancies, or patients affected by rheumatologic diseases treated with anti-TNF drugs). Signs and symptoms can be variables, depending upon involved organs. Symptoms of pulmonary localization commonly include cough, chest pain, fever and dyspnea. Diagnosis can be established by microscopic examination of sputum or bronchoalveolar lavage (BAL) specimens, serum antigen testing, and biopsy with culture. Chest radiographs usually show nodules with surrounding ground glass infiltrates (typical halo sign) (Figure 2). There is no indication for prophylaxis against IA among patients with immune-mediated inflammatory diseases treated with biologic DMARDs. The drug of choice for invasive infection is voriconazole, starting with a loading dose (6 mg/kg every 12 h for two doses) followed by maintenance doses (4 mg/kg every 12 h) for 6–12 weeks, or after resolution of symptoms. Alternative drugs are liposomal amphotericin B, posaconazole, itraconazole, isavuconazole and caspofungin. Anti-TNF therapy should be withhold while the infection is treated [4].

#### 4.2.3. Histoplasmosis

Histoplasmosis is a worldwide endemic mycosis caused by a fungus, *Histoplasma capsulatum*, particularly common in Central America and western areas of US. Transmission is related to environmental contamination (e.g., exposure to demolition of old buildings, spelunking, care of chickens). In immunocompetent patients, histoplasmosis usually presents as acute pneumonia with not-specific symptoms such as cough, dyspnea and fever. Disseminated disease occurs in approximately 1/2000 patients with acute infection, mainly with immunosuppression in AIDS, solid organ transplantation or treatment with anti-TNF agents. A chronic cavitary pulmonary form has also been reported. Histoplasmosis results to be the most common invasive fungal infection among patients treated with anti-TNF agents [4,95]. If histoplasmosis is suspected, a chest X-ray should be performed (usually showing interstitial pneumonitis or diffuse or localized infiltrates). Biopsy of pulmonary lesions or BAL may be necessary in some cases. If available, serum or urine antigen tests should be performed. If the tests are positive, the treatment with biological drugs should be stopped, and antifungal therapy with itraconazole (200–400 mg daily) for 6–12 weeks should be initiated. In case of severe or disseminated infections, a regimen of 7–14 days of amphotericin B (deoxycholate 0.7–1.0 mg/kg/day or liposomal 3–5 mg/kg/day) followed by itraconazole (200 mg twice daily) for 12 weeks is preferred. Central nervous system histoplasmosis resulted to be particularly difficult to treat, due to low penetration of most antifungal agents into the liquor. Methylprednisolone (0.5–1.0 mg/kg daily intravenously) during the first 1–2 weeks of antifungal therapy is recommended in case of respiratory complications, including hypoxemia or significant respiratory distress [98].

#### 4.2.4. Coccidioidomycosis

Coccidioides species are dimorphic fungi of the genus *Coccidioides* (*Coccidioides immitis* and *Coccidioides posadasii*), endemic in the southwestern US (California, Arizona, New Mexico, Texas), Mexico, and parts of Central and South America. The infection is transmitted by inhalation of arthroconidia released from the soil into the air. Each year, approximately 150,000 new infections are reported in the US [99]. In immunocompetent hosts, more than half of the infections are asymptomatic or induce a self-limited nonspecific pulmonary illness (chest pain, cough, and fever). Up to 5% of patients develop complicated pulmonary disease, and approximately 1% develop disseminated infection (mainly immunocompromised patients). Some studies show that approximately 29% of community-acquired pneumonia in the endemic regions may be due to coccidioidomycosis [100,101], as well as a significant part of pneumonia developed shortly after a travel to endemic areas. In endemic areas, patients treated with TNF inhibitors are at risk of developing coccidioidomycosis [102]. In 2004, a study reported, over a 3-year period, a cumulative incidence of coccidioidomycosis of 1% (11/985 patients) [103]. Pneumonia resulted to be the most common manifestation, with systemic dissemination in 25% of cases. New infections rather than reactivation of previously acquired infections were responsible of most cases. Chest radiograph may show a diffuse bilateral reticulonodular pattern, usually indistinguishable from *Pneumocystis* pneumonia. Coccidioidomycosis infection should be suspected in all patients travelling from or living in endemic areas. Culture and PCR on sputum or BAL specimen, serology, biopsy, and molecular test for coccidioidal DNA can be useful in diagnosis. Anticoccidioidal antibody detection does not readily differentiate between new and old infection and the sensibility can be impaired in immunocompromised patients. In case of severe infections, urine antigen may be detected. Real-time PCR for coccidioidal DNA in respiratory secretions, even if still not routinely used in clinical practice, shows 100% sensitivity and 98% specificity for the diagnosis when compared to culture. Before starting anti-TNF therapy in endemic areas, clinicians should consider serological tests (IgG and IgM) and a chest radiograph to establish baseline, even if usually the infection is not the result of a reactivation. When diagnosed, invasive pulmonary coccidioidomycosis should be treated with fluconazole at the dose of 400–800 mg/day for 3–6 months, and anti-TNF therapy should be discontinued. For severe systemic infections, treatment usually consists of amphotericin B deoxycholate (0.5–1 mg/kg/day) until clinical improvement is achieved, followed by fluconazole (400–800 mg/day) for 12 months [4,104]. Continuing or restarting biologic therapy after resolution of infection is feasible, depending on severity of the preceding infection [105].

### 4.3. Viral Infection

#### Cytomegalovirus Pneumonia

Cytomegalovirus (CMV) is a widespread virus, with primary exposure usually occurring at an early age. The seroprevalence of prior CMV infection ranges from 40% to 100% in adults, depending on geographical areas and life conditions. Adult-onset CMV disease may be the result of primary infection, reinfection or, most commonly, reactivation of a latent infection. The primary infection in immunocompetent patients is almost always asymptomatic, or it presents as a mild mononucleosis-like disease. Depending on host immune status, CMV disease can present as a potentially fatal infection with multi-organ involvement (e.g., pneumonia, colitis, retinitis, hepatitis, encephalitis) mostly in patients with acquired immunodeficiency syndrome (AIDS), in patients treated long-term with glucocorticoids or immunosuppressive drugs and in recipients of organ transplants [106]. In immunosuppressed patients, CMV infection can cause a variety of mild and nonspecific symptoms and laboratory findings (e.g., slight fever and a low increase in levels of hepatic enzymes, cytopenia) which can lead to diagnostic delay. The detection of anti-CMV IgM antibodies assesses recent CMV infection but sensitivity is not satisfactory in patients with impaired immune response or treated with immunosuppressive drugs. The more sensitive and specific determination of CMV DNA by PCR methods has replaced the assessment of CMV antigenemia in the diagnosis of current CMV infections. Chest radiography plays an important role in detection of pulmonary infiltration and in evaluation of response to therapy. Nevertheless, this exam results often nonspecific, with low sensitivity in case of early infections, particularly in immunocompromised patients. CT may be indicated in case of moderate or severe symptoms suggesting lung involvement such as cough, dyspnea, chest pain. In CMV pneumonia, chest high-resolution CT may document diffuse or patchy consolidation, small centrilobular nodules, ground-glass opacities, bronchial wall thickening, a combination of consolidation and reticular opacities and pleural effusion. Rare cases of cavitary lung lesions have been reported [107]. Demirkazik and collegues analyzed CT scans of immunosuppressed patients in order to assess typical features of opportunistic pulmonary infections. On chest CT scan, centrilobular nodules were the most frequent abnormality detectable (75% of patients), whereas consolidation was observed in the other 25% of patients. The centrilobular nodules were associated with bronchial wall thickening and ground-glass nodules or densities in the majority of cases of CMV pneumonia. Conversely, consolidation was more common in bacterial infections (68.4%) [108]. No PCR screening is recommended for CMV prior to starting anti-TNF therapy in asymptomatic patients. A careful examination for manifestations of infection prior to the initiation and during treatment is recommended and CMV DNA quantification should be performed if CMV infection is suspected. For patients affected by mild disease with low plasmatic viral load, no therapy is indicated, and anti-TNF therapy can be continued with a close monitoring. In case of severe infections, immunosuppressive therapy should be discontinued and antiviral treatment should be started. First treatment option is ganciclovir at the dose of 5 mg/kg IV twice daily for 14–21 days; after 3–5 days if clinical conditions allow it, it is possible to switch to oral therapy with valganciclovir. Alternatively, in cases of ganciclovir resistance or intolerance, foscarnet at the dose of 90 mg/kg IV twice daily or 60 mg/kg IV three times daily may be used [4].

## 5. Conclusions

**Figure 3:**
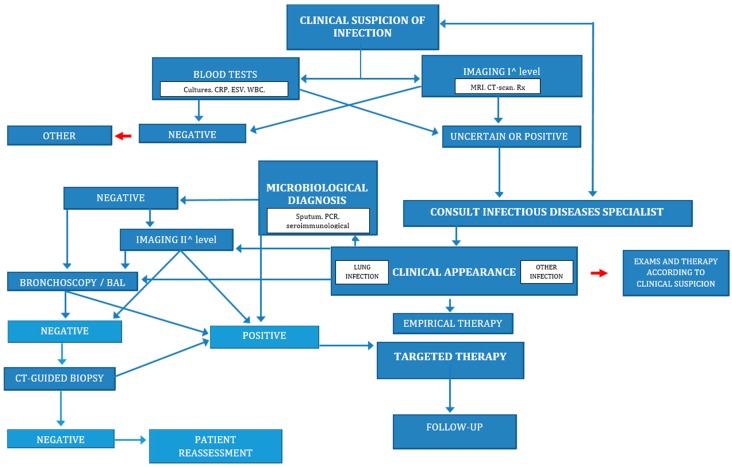
Diagnosis and management of lung infection in rheumatic diseases. CRP: c-reactive protein; ESV: erythrocyte sedimentation rate; WBC: white blood cells; MRI: magnetic resonance imaging; CT: computed tomography; Rx: radiography; PCR: polymerase chain reaction; BAL: bronchoalveolar lavage.

Despite a general increase of survival rate, infections remain a major cause of morbidity and mortality in rheumatic disease patients. The lung has been reported as the most frequent site of infections in rheumatic diseases. Although infections due to common bacteria are frequent and often severe, rheumatologic patients are predisposed also to opportunistic infections. Due to the lack of specificity of the clinical features in the early stages of infection, the physician should be always careful in patient assessment. In case of clinical suspicion of lung infection, all the necessary investigations must be promptly performed and appropriated therapy initiated (Figure 3). As a consequence of the complexity of these cases, a multidisciplinary approach is strongly recommended. A multidisciplinary team should include a rheumatologist and an infectious disease specialist skilled in management of patients treated with immunosuppressive drugs. Considering the potential high burden of mortality of infections in rheumatic patients, investigations must to be carried out quickly, avoiding diagnostic delay. A careful assessment of the patient, including BAL or lung biopsy when indicated, is often necessary to etiological diagnosis. The targeted treatments should be started as soon as possible.

## Figures and Tables

**Table 1:** Most frequent infections associated with immunosuppressive drugs.

Drug or Class of Drugs	Associated Infections
Glucocorticoid	High risk of pneumonia, skin infections and local candidiasis, increased incidence of opportunistic infections (mycobacterial, fungal and viral infections), VZV reactivation.
Methotrexate	Slight risk increase of pneumonia, urinary and skin infections and VZV reactivation.
Sulphasalazine	Slight increased incidence of septic arthritis.
Hydroxichloroquine	Favorable safety profile for infections.
Leflunomide	Moderate risk increase of higher and lower airways infections, cases of tuberculosis reported.
Cyclosporin	Moderate risk increase of pneumonia, increased incidence of opportunistic (mycotic, viral) infections.
Cyclofosfamide	High risk of bacterial respiratory, urinary, skin and sinus infections, bacteremia and septic shock, VZV reactivation, opportunistic mycotic and viral lung, CNS, esophageal infections.
Mycophenolate mofetil	Increased incidence of pneumonia, urinary infections, cellulitis and septic arthritis, increased incidence of viral infections (CMV and VZV reactivations).
Azathioprine	Increased incidence of pulmonary, urinary and skin bacterial and mycotic infections, VZV reactivation.
Anti-TNF	High risk of tuberculosis and non-tuberculous mycobacteria infections, respiratory, skin, soft-tissues and urinary infections, surgical site infections, opportunistic mycotic infections (*Histoplasma, Coccidioides, Pneumocystis, Aspergillus, Nocardia*), legionellosis, disseminated *Listeria* and *Salmonella* infections, VZV, HBV, HCV reactivation.
Tocilizumab	Pneumonia and pyogenic bacterial infections, diverticulitis and perforation, invasive aspergillosis and tuberculosis reported.
Rituximab	Pneumonia and pyogenic bacterial infections, PML, HBV reactivation, pneumocystosis, invasive aspergillosis and tuberculosis reported.
Abatacept	Pneumonia and pyogenic bacterial infections, invasive aspergillosis and tuberculosis reported.

Abbreviations: VZV: varicella-zoster virus; CNS: central nervous system; CMV: cytomegalovirus; HBV: hepatitis B virus; HCV: hepatitis C virus; PML: progressive multifocal leukoencephalopathy; TNF: tumor necrosis factor.
